# Upregulated Expression of MicroRNA-204-5p Leads to the Death of Dopaminergic Cells by Targeting DYRK1A-Mediated Apoptotic Signaling Cascade

**DOI:** 10.3389/fncel.2019.00399

**Published:** 2019-09-13

**Authors:** Ching-Chi Chiu, Tu-Hsueh Yeh, Rou-Shayn Chen, Hua-Chien Chen, Ying-Zu Huang, Yi-Hsin Weng, Yi-Chuan Cheng, Yu-Chuan Liu, Ann-Joy Cheng, Ya-Ching Lu, Yu-Jie Chen, Yan-Wei Lin, Chia-Chen Hsu, Ying-Ling Chen, Chin-Song Lu, Hung-Li Wang

**Affiliations:** ^1^Neuroscience Research Center, Chang Gung Memorial Hospital, Taoyuan, Taiwan; ^2^Healthy Aging Research Center, Chang Gung University College of Medicine, Taoyuan, Taiwan; ^3^Department of Nursing, Chang Gung University of Science and Technology, Taoyuan, Taiwan; ^4^Division of Movement Disorders, Department of Neurology, Chang Gung Memorial Hospital, Taoyuan, Taiwan; ^5^Department of Medical Biotechnology and Laboratory Science, College of Medicine, Chang Gung University, Taoyuan, Taiwan; ^6^Department of Neurology, Taipei Medical University Hospital, Taipei, Taiwan; ^7^School of Medicine, Taipei Medical University, Taipei, Taiwan; ^8^College of Medicine, Chang Gung University, Taoyuan, Taiwan; ^9^Genomic Core Laboratory, Molecular Medicine Research Center, Chang Gung University, Taoyuan, Taiwan; ^10^Institute of Cognitive Neuroscience, National Central University, Taoyuan, Taiwan; ^11^Graduate Institute of Biomedical Sciences, College of Medicine, Chang Gung University, Taoyuan, Taiwan; ^12^Division of Sports Medicine, Taiwan Landseed Hospital, Taoyuan, Taiwan; ^13^Department of Physiology and Pharmacology, Chang Gung University College of Medicine, Taoyuan, Taiwan

**Keywords:** Parkinson’s disease, microRNA-204-5p, DYRK1A, ER stress, autophagy, apoptotic signaling

## Abstract

MicroRNAs (miRs) downregulate or upregulate the mRNA level by binding to the 3′-untranslated region (3′UTR) of target gene. Dysregulated miR levels can be used as biomarkers of Parkinson’s disease (PD) and could participate in the etiology of PD. In the present study, 45 brain-enriched miRs were evaluated in serum samples from 50 normal subjects and 50 sporadic PD patients. The level of miR-204-5p was upregulated in serum samples from PD patients. An upregulated level of miR-204-5p was also observed in the serum and substantia nigra (SN) of 1-methyl-4-phenyl-1,2,3,6-tetrahydropyridine (MPTP) mouse model of PD. Expression of miR-204-5p increased the level of α-synuclein (α-Syn), phosphorylated (phospho)-α-Syn, tau, or phospho-tau protein and resulted in the activation of endoplasmic reticulum (ER) stress in SH-SY5Y dopaminergic cells. Expression of miR-204-5p caused autophagy impairment and activation of c-Jun N-terminal kinase (JNK)-mediated apoptotic cascade in SH-SY5Y dopaminergic cells. Our study using the bioinformatic method and dual-luciferase reporter analysis suggests that miR-204-5p positively regulates mRNA expression of dual-specificity tyrosine phosphorylation regulated kinase 1A (DYRK1A) by directly interacting with 3′UTR of DYRK1A. The mRNA and protein levels of DYRK1A were increased in SH-SY5Y dopaminergic cells expressing miR-204-5p and SN of MPTP-induced PD mouse model. Knockdown of DYRK1A expression or treatment of the DYRK1A inhibitor harmine attenuated miR-204-5p-induced increase in protein expression of phospho-α-Syn or phospho-tau, ER stress, autophagy impairment, and activation of JNK-mediated apoptotic pathway in SH-SY5Y dopaminergic cells or primary cultured dopaminergic neurons. Our results suggest that upregulated expression of miR-204-5p leads to the death of dopaminergic cells by targeting DYRK1A-mediated ER stress and apoptotic signaling cascade.

## Introduction

Parkinson’s disease (PD), the most common neurodegenerative motor disorder, is characterized by selective neuronal death of the substantia nigra (SN) pars compacta (SNpc) dopaminergic cells (Dickson et al., 2009). A pathological hallmark of PD is the accumulation of cytoplasmic inclusions, mainly consisting of alpha-synuclein (α-Syn) protein aggregates, in surviving SNpc neurons (Surmeier et al., 2017). While the precise pathogenic mechanism of PD is not clear, oxidative stress, mitochondrial malfunction, activation of apoptotic death cascade, endoplasmic reticulum (ER) stress, and autophagy/mitophagy impairment are believed to participate in the etiology of PD (Michel et al., 2016; Puspita et al., 2017; Remondelli and Renna, 2017; Zeng et al., 2018; Zhang et al., 2018).

MicroRNAs (miRs), small non-coding RNAs, modulate the expression of mRNA by interacting with the 3′-untranslated region (3′UTR) of the target gene (Bartel, 2004). In addition to posttranscriptional repression, miRs and miR-associated protein complexes also mediate the upregulation of gene expression (Vasudevan, 2012; Valinezhad Orang et al., 2014). Several studies reported that miRs regulate the level of PD genes, including *SNCA*, *LRRK2*, and *Parkin* (Arshad et al., 2017; Leggio et al., 2017; Martinez and Peplow, 2017; Singh and Sen, 2017). Moreover, miRs also participate in the regulation of neuronal development, ER stress, mitochondrial function, and autophagy (Arshad et al., 2017; Lu et al., 2017; Singh and Sen, 2017). The expressions of miRs exhibit cell and tissue specificity (Lee et al., 2008; Ludwig et al., 2016). Many brain-enriched miRs have been identified and can be detected in body fluids, such as serum and plasma and cerebrospinal fluid, from PD patients (Nelson et al., 2008; Lu et al., 2017; Sheinerman et al., 2017). Dysregulated levels of miRs can be used for biomarkers of PD and are believed to participate in the etiology of PD (Lu et al., 2017; Ramaswamy et al., 2018; Roser et al., 2018).

In the present study, we evaluated the level of brain-enriched miRs in serum samples from healthy subjects and sporadic PD patients. Our study indicated that the level of miR-204-5p was increased in serum samples from PD patients and in the SN of 1-methyl-4-phenyl-1,2,3,6-tetrahydropyridine (MPTP)-treated PD mouse model. Our results further suggest that the upregulated level of miR-204-5p increases the mRNA and protein expression levels of dual-specificity tyrosine phosphorylation regulated kinase 1A (DYRK1A).

DYRK1A participates in regulating neurogenesis, neuronal functions, cell survival, and apoptotic cell death (Choi and Chung, 2011; Tejedor and Hammerle, 2011; Kay et al., 2016). DYRK1A phosphorylates numerous neurodegenerative disorder-related proteins, including tau and α-Syn, and causes the accumulation of these proteins (Kim et al., 2006; Ryoo et al., 2007). The upregulated level of DYRK1A is believed to participate in the etiology of neurodegenerative disorders, including Alzheimer’s disease (AD), PD, and Huntington’s disease (HD) (Kang et al., 2005; Abbassi et al., 2015; Kay et al., 2016). In the present study, our data suggest that an increased level of miR-204 results in the death of dopaminergic cells by upregulating the expression of DYRK1A and targeting the DYRK1A-mediated apoptotic signaling pathway.

## Materials and Methods

### Participants and Collection of Serum Samples

Fifty patients affected with sporadic PD and 50 healthy control subjects were enrolled from Department of Neurology, Chang Gung Memorial Hospital. This study was reviewed and approved by the Institutional Review Board of Chang Gung Memorial Hospital (IRB no. 201601684B0), and written informed consent was provided by all the subjects. The clinical diagnosis of PD was confirmed as described previously (Gelb et al., 1999). The demographic information was listed in Supplementary Table 1. The mean age of the healthy controls was not significantly different from that of the PD patients (Supplementary Table 1). Blood specimens were collected in 10-ml BD Vacutainer glass tubes without additive (BD 367985, catalog no. 02-683-98, BD Biosciences) and coagulated at 25°C. Following the centrifugation, serum samples were obtained and aliquoted.

### Extraction of miRs and Real-Time Quantitative Reverse Transcription Polymerase Chain Reaction (qRT-PCR) Analysis

The miRs were obtained from human serum samples, SH-SY5Y cells, or SN tissues of mice by using miRNeasy Serum/Plasma Kit (Qiagen) or miRNeasy Mini Kit (Qiagen). The levels of brain-enriched miRs were examined by stem-loop RT-PCR according to a previous study (Chen et al., 2009). Briefly, 0.1 μg of total RNA from serum samples or 0.5 μg of total RNA from SH-SY5Y dopaminergic neurons or mouse SN tissue was added to the RT reaction reagent containing miR-specific RT primers. The RT reactions of miRs were processed with the program: 16°C for 30 min, 50 cycles at 20°C for 30 s, 42°C for 30 s, and 50°C for 1 s. ABI Prism 7900 Fast Real-Time PCR system was used to determine the levels of miRs with the universal reverse primer and miR-specific forward primers. For miR quantification, raw threshold cycle (Ct) values were transformed to 39-Ct and normalized with total average or U6 snRNA to analyze the expression of miR (Chen et al., 2009). Each quantitative RT-PCR experiment was performed in triplicate.

### Preparation of mRNA and qRT-PCR Analysis

The miRNeasy Mini Kit was used to obtain total RNA from SH-SY5Y dopaminergic cells or mouse SN. Briefly, 0.5 μg of total RNA from SH-SY5Y dopaminergic cells and mouse SN tissue was added to an RT reaction mixture containing oligo-dT primers. The RT reaction of mRNA was conducted at 50°C for 60 min and terminated at 70°C for 15 min. For the quantification of mRNA, PCR reaction was conducted for 40 times with the following program: 95°C for 10 s and 60°C for 30 s. For mRNA expression of target genes, GAPDH housekeeping gene was used to normalize mRNA level. The relative level of mRNA was calculated with the 2^–(ΔΔCt)^ equation. Each qRT-PCR experiment was performed in triplicate.

### MPTP-Treated Mouse Model of PD

The animal study was carried out in accordance with the principles of the Basel Declaration and the guideline of the Institutional Animal Care and Use Committee of Chang Gung University. The protocol was reviewed and approved by the Institutional Animal Care and Use Committee of Chang Gung University (no. CGU16-047). Twelve-month-old male C57BL/6 (B6) mice were intraperitoneally treated with saline or MPTP (Sigma) with a daily dose of 20 mg/kg for 14 days (Chiu et al., 2015). Then, mouse SN was excised for immunoblotting or qRT-PCR analysis. As described previously (Chiu et al., 2015), MPTP treatment caused neurodegeneration of SNpc dopaminergic cells.

### Cell Culture and Transfection

SH-SY5Y dopaminergic neuron-like cells (Constantinescu et al., 2007; Lopes et al., 2010; Xie et al., 2010; Korecka et al., 2013; Xicoy et al., 2017; Avola et al., 2018; Taylor-Whiteley et al., 2019; Titze-de-Almeida et al., 2019; Wiedmer et al., 2019) were cultured in Dulbecco’s modified Eagle’s medium (DMEM)/F12 medium supplemented with 10% fetal bovine serum. SH-SY5Y dopaminergic cells or primary cultured dopaminergic neurons were transfected with miR-204-5p mimic (5′-UUCCC UUUGUCAUCCUAUGCCU-3′), scramble miR control (SC, 5′-GUCCUCUUUCACCCGUUUCUUA-3′), or shRNAs of DYRK1A (sequence of shRNA1:CCGGGTTCGGCTTGCACCG TCATTTCTCGAGAAATGACGGTGCAAGCCGAACTTTTTG; sequence of shRNA2: CCGGGTTCGGCTTGCACCGTCATTTCT CGAGAAATGACGGTGCAAGCCGAACTTTTTG). For inhibition of DYRK1A activity, SH-SY5Y cells or primary cultured dopaminergic neurons were treated with 1 μM harmine, the specific DYRK1A inhibitor (Gockler et al., 2009; Kumar et al., 2018), for 24 h.

### Preparation of Primary Cultured Dopaminergic Neurons

Primary cultured SN dopaminergic neurons were prepared according to our methods described previously (Wang et al., 2011; Chiu et al., 2015). Briefly, SN was dissected from embryonic day 15 to 17 mice and incubated in DMEM/F12 medium containing 0.3 mg/ml DNase I and 0.5 mg/ml Pronase at 37°C for 50 min. SN tissues were subsequently triturated by using the Pasteur pipette. Dissociated cells were plated on the poly-L-lysine-coated dish and cultured with DMEM/F12 medium supplemented with 5% fetal bovine serum, 5% horse serum, GDNF (glial cell line-derived neurotrophic growth factor; 25 ng/ml), 5′-fluoro-2′-deoxyuridine, and uridine. Large multipolar or oval-shaped dopaminergic neurons (diameter = 25–30 μm) were observed in primary SN neuronal culture. About 40% of cultured neurons were dopaminergic cells. Seven-day-old primary cultured dopaminergic neurons were used for the experiments.

### Analysis of Cell Viability

Cell Counting Kit-8 assay was conducted to examine the survival of SH-SY5Y dopaminergic cells or primary cultured dopaminergic neurons transfected with miR-204-5p mimic, SC, or shRNA of DYRK1A. Each experiment was performed in triplicate.

### Identification and Validation of miR-204-5p Target Genes

Target genes of miR-204-5p were identified using TargetScan, miRBase, and PicTar database. Dual-luciferase reporter analysis was performed to study the binding between miR-204-5p and target genes. According to TargetScan and PicTar database, DYRK1A is a predicted target of miR-204-5p. The 3′UTR of DYRK1A gene (2.8 kb) containing seed regions of miR-204-5p was subcloned into pmirGLO dual-luciferase miRNA Expression Vector (p-3′UTR-DYRK1A). The binding between miR-204-5p and 3′UTR of DYRK1A was evaluated by using pmirGLO dual-luciferase miRNA Expression Vector containing wild-type (WT) (WT 3′UTR-DYRK1A) or mutant 3′UTR of DTRK1A (Mut 3′UTR-DYRK1A). WT 3′UTR-DYRK1A or Mut 3′UTR-DYRK1A and scramble miR control or miR-204-5p mimic were cotransfected into SH-SY5Y cells. Two days after the transfection, the luciferase activity was examined by using the dual-luciferase reporter assay system. Each experiment was performed in triplicate.

### Western Blotting

SH-SY5Y dopaminergic cells, mouse SN, or primary cultured dopaminergic neurons were homogenized in CHAPS buffer containing protease inhibitors. Proteins (30□μmg) of SH-SY5Y dopaminergic cells, mouse SN, or primary cultured dopaminergic neurons were separated on SDS-polyacrylamide gel and then transferred onto nitrocellulose membranes. Subsequently, membranes were interacted with following primary antibodies: (1) anti-DYRK1A antibody (Cat# H00001859-M01, clone 7D10, RRID: AB_534844) from Abnova; (2) anti-α-Syn antiserum (Cat# 10842-1-AP, RRID: AB_2192672) from Proteintech; (3) anti-phosphorylated (phospho)-α-Syn^Ser129^ antibody (Cat# ab51253, RRID: AB_869973) from Abcam; (4) anti-tau antiserum (Cat# sc-32274, RRID: AB_628327) from Santa Cruz; (5) anti-phospho-tau^Ser202/Thr205^ antiserum (Cat# MN1020, RRID: AB_223647) from Thermo Fisher Scientific; (6) anti-actin antibody (Cat# MAB1501, RRID: AB_2223041) from Merck; (7) anti-glucose regulated protein78 (Grp78) antiserum (Cat# 3177, RRID: AB_2119845) from Cell Signaling Technology; (8) anti-protein kinase RNA-like ER kinase (PERK) antibody (Cat# 5683, RRID: AB_10841299) from Cell Signaling Technology; (9) anti-C/EBP homologous protein (CHOP) antiserum (Cat# 2895, RRID: AB_2089254) from Cell Signaling Technology; (10) anti-Beclin-1 antibody (Cat# 3495, RRID: AB_1903911) from Cell Signaling Technology; (11) anti-autophagy-related protein 7 (Atg7) antiserum (Cat# 8558, RRID: AB_10831194) from Cell Signaling Technology; (12) anti-autophagy-related protein 16-1 (Atg16L1) antibody (Cat# 8089, RRID: AB_10950320) from Cell Signaling Technology; (13) anti- microtubule-associated protein 1A/1B-light chain 3 (LC3A/B) antiserum (Cat# 12741, RRID: AB_2617131) from Cell Signaling Technology; (14) anti-caspase-12 antibody (Cat# 2202, RRID: AB_2069200) from Cell Signaling Technology; (15) anti-cleaved active caspase-9 antiserum (Cat# 9964, RRID: AB_2070042) from Cell Signaling Technology; (16) anti-cleaved active caspase-3 antibody (Cat# 9662, RRID: AB_331439) from Cell Signaling Technology; (17) anti-c-Jun N-terminal kinase (JNK) antiserum (Cat# 9252, RRID: AB_2250373) from Cell Signaling Technology; (18) anti-phospho-JNK^Thr183/Tyr185^ antibody (Cat# 4668, RRID: AB_823588) from Cell Signaling Technology; (19) anti-c-Jun antiserum (Cat# 9165, RRID: AB_2130165) from Cell Signaling Technology; (20) anti-phospho-c-Jun^Ser73^ antibody (Cat# 3270, RRID: AB_2129575) from Cell Signaling Technology; and (21) anti-inositol-requiring enzyme 1α (IRE1α) (Cat# 3294, RRID: AB_823545) antiserum from Cell Signaling Technology. Membranes were washed and probed with secondary horseradish peroxidase (HRP)-conjugated antibodies. Chemiluminescence reagents were used to visualize immunoreactive signal. The value of protein expression was determined with the densitometer and normalized to the signal of actin.

### Statistical Analysis

SPSS software and GraphPad Prism software were used to examine statistical analysis. Data were presented as mean ± standard error of the mean (SEM). The significant difference between two groups was determined with unpaired Student’s *t*-test (two-tailed). One-way analysis of variance (ANOVA) and Tukey test were performed to analyze significant differences among multiple study groups. The *p*-value less than 0.05 was indicated as statistical significance.

## Results

### The Level of miR-204-5p Is Increased in Serum Samples From Sporadic PD Patients

**FIGURE 1:**
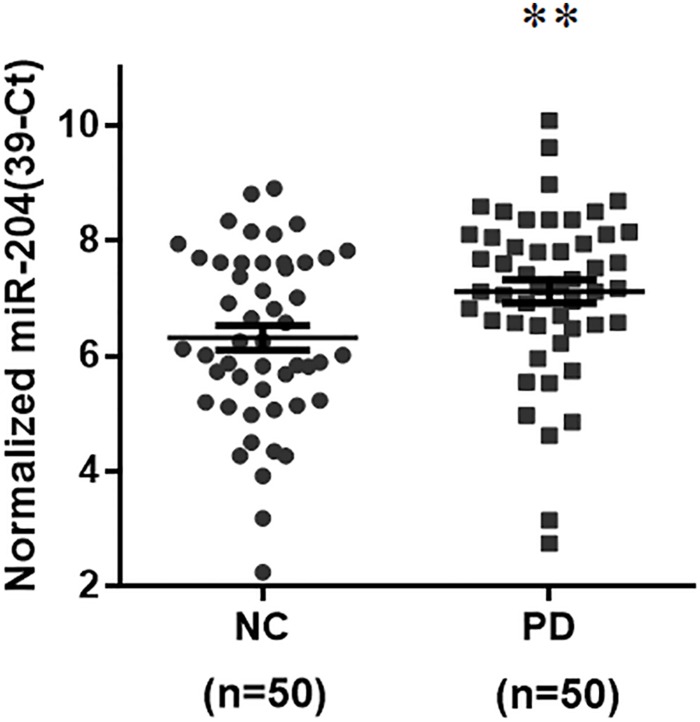
Expression of miR-204-5p is upregulated in serum samples from sporadic PD patients. The qRT-PCR assays were performed to analyze the expression level of brain-enriched miRs in serum samples from PD patients. The relative level of miR-204-5p in NC (*n* = 50) was 6.32 ± 0.21 (mean ± SEM). The relative expression of miR-204-5p in PD patients (*n* = 50) was 7.13 ± 0.20 (mean ± SEM). Note that the serum level of miR-204-5p was significantly increased in PD patients. The relative level of miR-204-5p was shown as 39-Ct following normalization. Each qRT-PCR experiment was performed in triplicate. ^∗∗^*p* < 0.01 compared with normal control (NC) subjects.

To identify a differentially expressed miR biomarker of PD that is detectable in the serum, the levels of 45 brain-enriched miRs (Supplementary Table 2) mentioned in a previous study (Kim et al., 2007) were evaluated in serum samples from 50 normal subjects and 50 sporadic PD patients (Supplementary Table 1). The qRT-PCR assays indicated that compared to serum samples from healthy subjects, the expression level of only one miR, miR-204-5p, was significantly increased in serum samples from PD patients (Figure 1). The serum level of miR-26a-5p, miR-138-5p, or miR-218-5p was significantly reduced in PD patients (Supplementary Table 2). In this study, we further studied the pathogenic mechanism of upregulated miR-204-5p.

### The Level of miR-204-5p Is Upregulated in the SN and Serum of MPTP Mouse Model of PD

**FIGURE 2:**
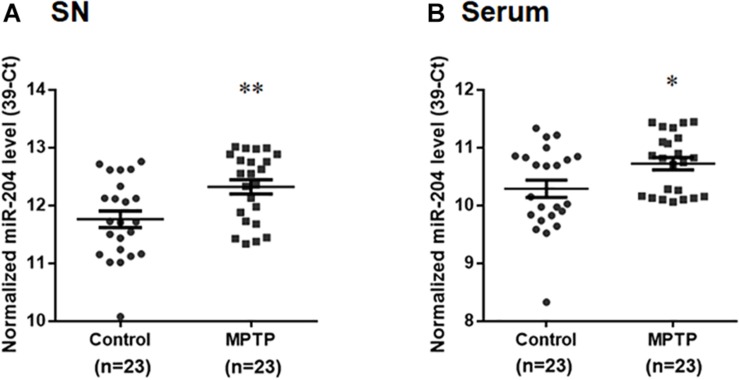
The level of miR-204-5p is upregulated in the SN and serum of the MPTP mouse model of PD. The expression level of miR-204-5p was evaluated in the SN or serum of MPTP-treated mice by performing qRT-PCR assay. **(A)** The relative level of miR-204-5p in control mice (*n* = 23) was 11.76 ± 0.14 (mean ± SEM). The relative expression of miR-204-5p in the MPTP-treated mice (*n* = 23) was 12.32 ± 0.12 (mean ± SEM). The expression of miR-204-5p was upregulated in the SN of the MPTP mouse model of PD. **(B)** Compared to control mice, the serum level of miR-204-5p was increased in the MPTP-treated mice. The relative level of miR-204-5p was indicated as 39-Ct following normalization. Each qRT-PCR experiment was performed in triplicate. ^∗^*p* < 0.05, ^∗∗^*p* < 0.01 compared with control mice.

To explore whether upregulated expression of miR-204-5p is involved in the pathogenesis of PD, we evaluated the level of miR-204-5p in the SN of MPTP mouse model of PD (Chiu et al., 2015). Compared to control mouse, the expression level of miR204 was upregulated in the SN of MPTP-treated mouse (Figure 2A). The level of miR-204-5p was further examined in serum samples from the MPTP mouse model of PD. Compared to control mice, the serum level of miR-204-5p was also significantly upregulated in the MPTP-treated mice (Figure 2B).

### Expression of miR-204-5p Upregulates the Protein Level of α-Syn, Phospho-α-Syn, Tau, or Phospho-Tau and Causes ER Stress in SH-SY5Y Dopaminergic Cells

**FIGURE 3:**
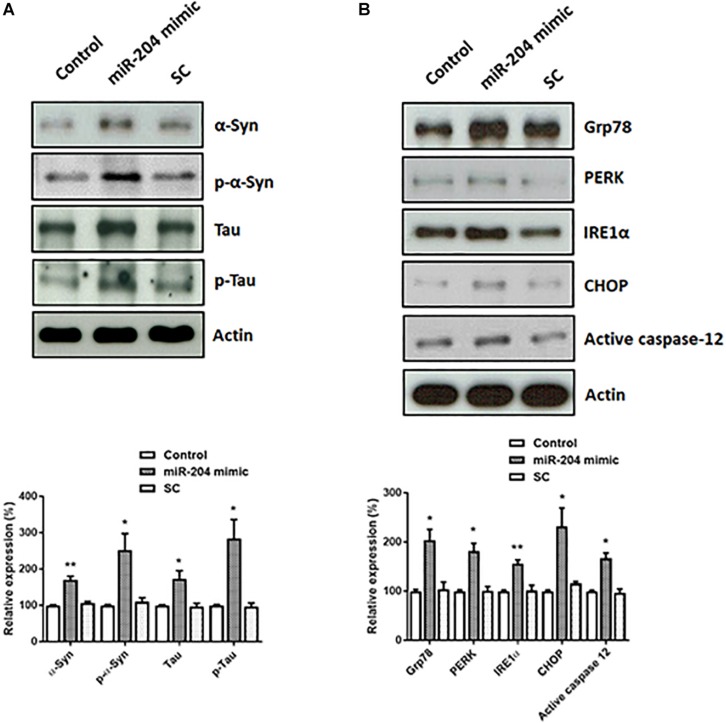
Expression of miR-204-5p in SH-SY5Y dopaminergic cells increases the protein levels of α-Syn, phospho-α-Syn, tau, or phospho-tau, and causes ER stress. **(A)** SH-SY5Y dopaminergic cells were transfected with the miR-204-5p mimic or scramble miR control (SC). Immunoblotting study demonstrated that the upregulated protein expression of α-Syn, phospho-α-SynSer129, tau, and phospho-tauSer202/Thr205 was observed in the miR-204-5p mimic-transfected SH-SY5Y dopaminergic cells. **(B)** Expression of miR-204-5p increased the protein levels of ER stress-related proteins, including Grp78, PERK, IRE1α, CHOP, and active caspase-12, in SH-SY5Y dopaminergic cells. The protein expression of actin was used as the loading control and the normalization control. Each bar shows the mean ± SEM value of four independent experiments. ^∗^*p* < 0.05, ^∗∗^*p* < 0.01 compared with control cells.

Lewy bodies are the molecular feature of PD and cause neurotoxicity (Spires-Jones et al., 2017; Sweeney et al., 2017; Wemheuer et al., 2017). Lewy bodies contain α-Syn, phospho-α-Syn, tau, and phospho-tau (Ishizawa et al., 2003). To investigate the role of upregulated miR-204-5p in the pathogenesis of PD, miR-204-5p scramble miR control (SC) or mimic was transfected to SH-SY5Y dopaminergic cells. Western blotting study indicated that compared to control or scramble miR control-transfected SH-SY5Y cells, expression of miR-204-5p significantly upregulated the protein expression of α-Syn, phospho-α-Syn^Ser129^, tau, or phospho-tau^Ser202/Thr205^ in SH-SY5Y dopaminergic cells (Figure 3A).

An increased protein level of α-Syn and phospho-α-Syn caused by miR-204-5p could lead to activation of ER stress (Wang and Hay, 2015; Ogen-Shtern et al., 2016; Ganguly et al., 2018). In accordance with our hypothesis, protein expressions of Grp78, IRE1α, PERK, and CHOP, which activate ER stress, were significantly upregulated in miR-204-5p mimic-transfected SH-SY5Y dopaminergic cells (Figure 3B). Expression of miR-204-5p in SH-SY5Y neurons also upregulated the protein level of ER stress-specific active caspase-12 (Figure 3B).

### Expression of miR-204-5p Causes Autophagy Impairment and the Activation of JNK-Mediated Apoptotic Cascade in SH-SY5Y Dopaminergic Cells

**FIGURE 4:**
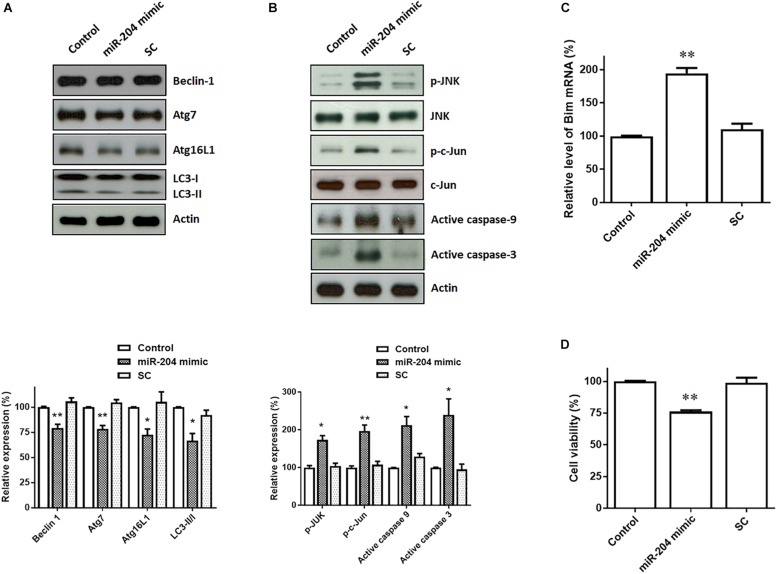
Expression of miR-204-5p in SH-SY5Y dopaminergic cells impairs autophagy and causes the activation of JNK-mediated apoptotic pathway. **(A)** Western blotting showed that expression of the miR-204-5p mimic resulted in the decrease in autophagy markers, including Beclin-1, Atg7, Atg16L1, and LC3-II/I ratio, in SH-SY5Y dopaminergic cells. **(B)** The protein expression of active phospho-JNKThr183/Tyr185 or phospho-c-JunSer63 was upregulated in the miR-204-5p mimic-transfected SH-SY5Y neurons. Expression of miR-204-5p led to the activation of caspase-9 and caspase-3 in SH-SY5Y dopaminergic cells. The protein expression of actin was used as the loading control and the normalization control. **(C)** qRT-PCR analysis showed that the mRNA level of Bim was significantly upregulated in SH-SY5Y neurons expressing miR-204-5p mimic. Each qRT-PCR experiment was performed in triplicate. **(D)** Expression of miR-204-5p caused a significant cell death of SH-SY5Y dopaminergic cells. For the assays of cell viability, each experiment was performed in triplicate. Each bar shows the mean ± SEM value of four independent experiments. ^∗^*p* < 0.05, ^∗∗^*p* < 0.01 compared with control cells.

Upregulated expression of α-Syn and phospho-α-Syn caused by miR-204-5p is expected to lead to the impairment of autophagic flux, which promotes ER stress (Winslow et al., 2010). Consistent with this hypothesis, immunoblotting analysis showed that protein levels of autophagy markers, such as Beclin-1, Atg7, Atg16L1), and LC3 ratio (LC3-II/I), were downregulated in SH-SY5Y dopaminergic cells expressing miR-204-5p (Figure 4A).

Jun N-terminal kinase pathway is induced by ER stress and leads to activation of neuronal apoptotic cascade (Peng and Andersen, 2003; Kuan and Burke, 2005). During induction of ER stress, IRE1, an ER sensor protein, activates downstream targets, JNK, and transcription factor c-Jun. Activated c-Jun causes the induction of active caspase-9 and active caspase-3 by upregulating the level of Bim mRNA. Compared to control or scramble miR control-transfected SH-SY5Y cells, expression of miR-204-5p mimic significantly upregulated the protein level of active phospho-JNK^Thr183/Tyr185^ or phospho-c-Jun^Ser63^ in SH-SY5Y dopaminergic cells (Figure 4B). The level of Bim mRNA was increased in miR-204-5p mimic-transfected SH-SY5Y dopaminergic cells (Figure 4C). Moreover, expression of miR-204-5p mimic led to the activation of caspase-9 and caspase-3 in SH-SY5Y dopaminergic cells (Figure 4B).

Activation of caspase-12, caspase-9, and caspase-3 caused by miR-204-5p could result in the death of SH-SY5Y dopaminergic cells. In accordance with this hypothesis, expression of miR-204-5p significantly reduced the viability of SH-SY5Y dopaminergic cells (Figure 4D).

### DYRK1A Is the Direct Target of miR-204-5p

**FIGURE 5:**
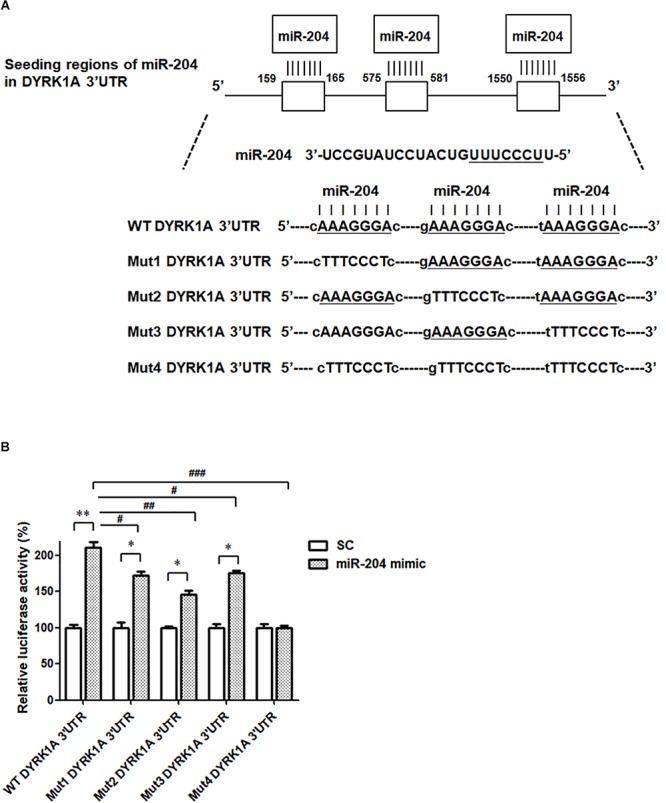
The miR-204-5p directly targets the 3′UTR of DYRK1A. **(A)** WT DYRK1A 3′UTR contains three predicted binding regions of miR-204-5p. Mut1, Mut2, Mut3, or Mut4 DYRK1A 3′UTR was prepared for analyzing the interaction between miR-204-5p and DYRK1A 3′UTR. **(B)** Dual-luciferase reporter assay was used to evaluate the interaction between miR-204-5p and DYRK1A 3′UTR. SH-SY5Y cells were cotransfected with reporter vector containing WT or mutant DYRK1A 3′UTR along with scramble miR control (SC) or miR-204-5p mimic. The level of luciferase activity was significantly increased in SH-SY5Y cells cotransfected with WT DYRK1A 3′UTR and miR-204-5p mimic. Compared with SH-SY5Y dopaminergic cells cotransfected with WT DYRK1A 3′UTR and miR-204-5p mimic, the luciferase activity of SH-SY5Y cells cotransfected with Mut1, Mut2, or Mut3 DYRK1A 3′UTR and miR-204-5p mimic was decreased. The miR-204-5p mimic failed to affect the luciferase activity of SH-SY5Y cells cotransfected with Mut4 DYRK1A 3′UTR. For dual-luciferase reporter assays, each experiment was performed in triplicate. Each bar represents the mean ± SEM value of four independent experiments. ^∗^*p* < 0.05, ^∗∗^*p* < 0.01 compared with scramble miR control (SC)-transfected SH-SY5Y neurons expressing WT or mutant DYRK1A 3′UTR. ^#^*p* < 0.05, ^##^*p* < 0.01, ^###^*p* < 0.001 compared with SH-SY5Y cells cotransfected with WT DYRK1A 3′UTR and miR-204-5p mimic.

To investigate the pathogenic mechanism underlying miR-204-5p-induced neurotoxicity, the bioinformatic method was further performed to predict the target genes of miR-204-5p. The miRs target the seed region of the 3′UTR of target gene and then regulate mRNA level. To identify the target gene of miR-204-5p, TargetScan, miRbase, and PicTar bioinformatic databases were used to predict the candidate target gene of miR-204-5p. Our analysis suggests that DYRK1A is the candidate target of miR-204-5p and that 3′UTR of DRYK1A contains three binding sites of miR204-5p (Figure 5A).

To examine whether miR-204-5p directly targets the 3′UTR of DYRK1A, WT 3′UTR of DYRK1A or mutant 3′UTR of DYRK1A (Mut1, Mut2, Mut3, or Mut 4) containing mutated seed region(s) (Figure 5A) was subcloned into the miRNA expression vector with dual luciferase. The upregulated level of luciferase activity was observed in SH-SY5Y cells cotransfected with WT DYRK1A 3′UTR and miR-204-5p mimic (Figure 5B). This finding suggests that miR-204-5p positively regulates mRNA expression of DYRK1A. The miR-204-5p mimic-induced increase in luciferase activity was impaired in SH-SY5Y cells transfected with Mut1, Mut2, or Mut3 DYRK1A 3′UTR (Figure 5B). Upregulation in luciferase activity caused by the miR-204-5p mimic was absent in SH-SY5Y cells transfected with Mut4 DYRK1A 3′UTR (Figure 5B). Our results suggest that miR-204-5p positively regulates the mRNA level of DYRK1A by directly interacting with the seed regions of the 3′UTR of DYRK1A.

### The Level of DYRK1A mRNA and DYRK1A Protein Is Increased in SH-SY5Y Dopaminergic Cells Expressing miR-204-5p and SN of the MPTP PD Mouse Model

**FIGURE 6:**
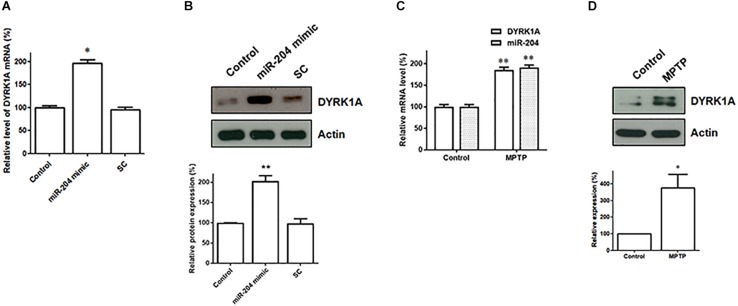
The mRNA and protein expression of DYRK1A is upregulated in SH-SY5Y dopaminergic cells expressing miR-204-5p and SN of the MPTP mouse model of PD. **(A)** Compared to control- or scramble miR control (SC)-transfected SH-SY5Y cells, the level of DYRK1A mRNA was significantly increased in SH-SY5Y dopaminergic cells transfected with the miR-204-5p mimic. Each qRT-PCR experiment was performed in triplicate. **(B)** An increased expression of DYRK1A protein was observed in the miR-204-5p mimic-transfected-SH-SY5Y cells. **(C)** An increased level of miR-204-5p in the SN of MPTP-treated mice was accompanied by an upregulated expression of DYRK1A mRNA in the SN of the MPTP mouse model of PD. Each qRT-PCR experiment was performed in triplicate. **(D)** The protein level of DYRK1A was significantly increased in the SN of MPTP-treated mice. Each bar indicates the mean ± SEM value of four independent experiments. ^∗^*p* < 0.05, ^∗∗^*p* < 0.01 compared with control.

Our data suggest that miR-204-5p positively regulates the mRNA expression of DYRK1A by directly interacting with the seed regions of the 3′UTR of DYRK1A (Figure 5). The levels of DYRK1A mRNA and DYRK1A protein were determined in SH-SY5Y dopaminergic cells expressing scramble miR control or miR-204-5p mimic. Compared to control-transfected SH-SY5Y cells, the expression of miR-204-5p resulted in an upregulated mRNA level of DYRK1A (Figure 6A). Moreover, an upregulated expression of the DYRK1A protein was observed in SH-SY5Y cells transfected with the miR-204-5p mimic (Figure 6B).

The level of miR204-5p was upregulated in the SN of MPTP-treated mice (Figure 2A). As a result, the expression of DYRK1A mRNA and DYRK1A protein is expected to be upregulated in the SN of PD MPTP mouse model. Consistent with this hypothesis, the mRNA level of DYRK1A was significantly increased in the SN of MPTP-treated mice (Figure 6C). The upregulated expression of the DYRK1A protein was also observed in the SN of the MPTP PD mouse model (Figure 6D).

### Knockdown of DYRK1A Expression Reverses miR-204-5p-Induced Increase in Protein Expression of Phospho-α-Syn or Phospho-Tau, ER Stress, Autophagy Impairment, and Activation of JNK-Mediated Apoptotic Pathway

In the present study, our results demonstrated that miR-204-5p caused the upregulated expression of phospho-α-Syn or phospho-tau, ER stress, autophagy impairment, and activation of JNK-mediated apoptotic cascade in SH-SY5Y dopaminergic cells. Our data also showed that miR-204-5p upregulated the level of DYRK1A mRNA and DYRK1A protein in SH-SY5Y dopaminergic cells. Previous studies reported that DYRK1A causes the phosphorylation of α-Syn or tau and the activation of JNK signaling (Kim et al., 2006; Ryoo et al., 2007; Choi and Chung, 2011). Therefore, it is very likely that miR-204-5p induces an increase in protein expression of phospho-α-Syn or phospho-tau, ER stress, autophagy impairment, and activation of JNK-mediated apoptotic cascade by increasing the mRNA and protein expression of DYRK1A. To test this hypothesis, shRNAs of DYRK1A, the DYRK1A inhibitor harmine, and the miR-204-5p mimic were cotransfected into SH-SY5Y dopaminergic cells and primary cultured SN dopaminergic neurons.

**FIGURE 7:**
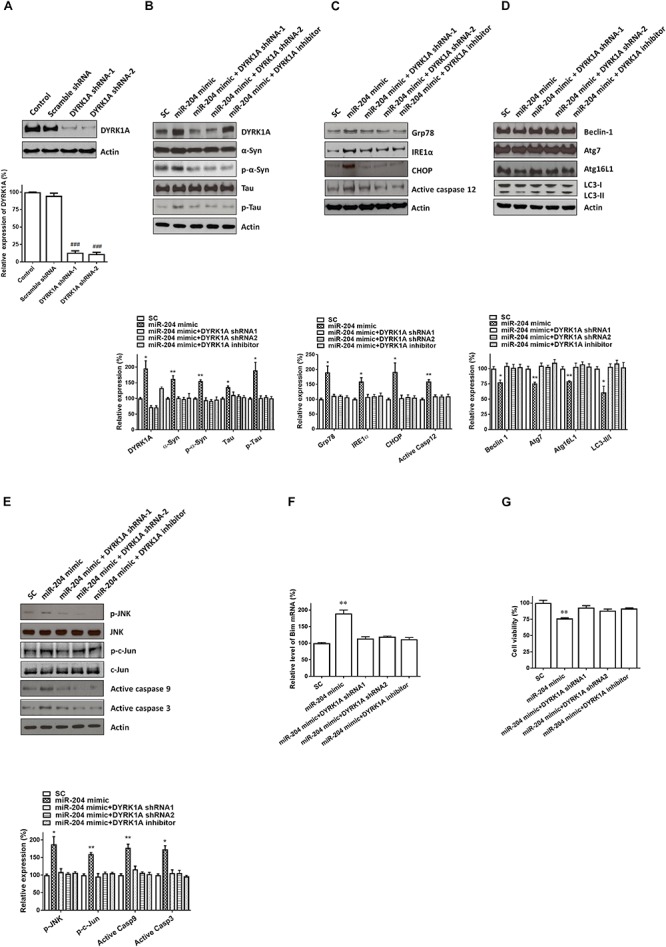
The miR-204-5p-induced neurotoxicity of SH-SY5Y dopaminergic cells is mediated by the upregulated expression of the DYRK1A protein. **(A)** Transfecting SH-SY5Y cells with shRNAs of DYRK1A greatly decreased the protein level of DYRK1A. Transfection of scramble control (Scramble) DYRK1A shRNAs did not affect the protein expression of DYRK1A. **(B)** In the presence of DYRK1A shRNAs and DYRK1A inhibitor harmine (1 μM), the miR-204-5p mimic failed to increase the protein expression of α-Syn, phospho-α-Syn, tau, or phospho-tau in SH-SY5Y dopaminergic cells. **(C)** Cotransfection of shRNAs of DYRK1A or treatment of DYRK1A inhibitor harmine reversed the miR-204-5p mimic-induced upregulation of ER stress-related proteins, including Grp78, IRE1α, CHOP, and active caspase-12, in SH-SY5Y cells. **(D)** Knockdown of DYRK1A expression in SH-SY5Y cells or harmine treatment attenuated miR-204-5p mimic-induced decrease in protein expression of autophagy markers, including Beclin-1, Atg7, Atg16L1, and LC3-II/I ratio. **(E)** The miR-204-5p mimic-induced activation of JNK, c-Jun, caspase-9, and caspase-3 in SH-SY5Y cells was blocked by cotransfection of the shRNA of DYRK1A or treatment of DYRK1A inhibitor harmine. The protein expression of actin was used as the loading control and the normalization control. **(F)** Cotransfection of DYRK1A shRNA or treatment of DYRK1A inhibitor harmine reversed miR-204-5p mimic-induced upregulation of Bim mRNA in SH-SY5Y dopaminergic cells. Each qRT-PCR experiment was performed in triplicate. **(G)** In the presence of DYRK1A shRNA or DYRK1A inhibitor harmine, the miR-204-5p mimic did not cause significant cell death of SH-SY5Y dopaminergic cells. For the assays of cell viability, each experiment was performed in triplicate. Each bar indicates the mean ± SEM value of four independent experiments. ^###^*p* < 0.001, compared to control SH-SY5Y neurons. ^∗^*p* < 0.05, ^∗∗^*p* < 0.01 compared to scramble miR control (SC)-transfected SH-SY5Y neurons.

**FIGURE 8:**
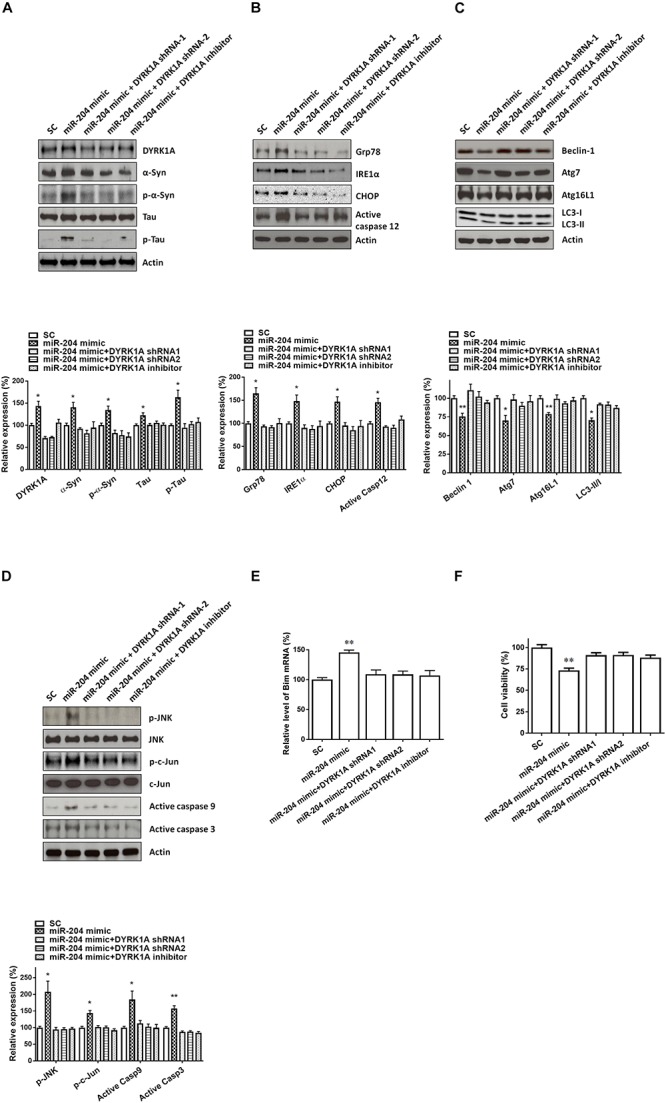
Neurotoxicity of primary cultured SN dopaminergic neurons caused by miR-204-5p is mediated by the increased level of DYRK1A protein. **(A)** Cotransfection of shRNAs of DYRK1A or treatment of DYRK1A inhibitor harmine (1μM) attenuated miR-204-5p mimic-induced upregulation of α-Syn, phospho-α-Syn, tau, or phospho-tau in primary cultured dopaminergic neurons. **(B)** In the presence of DYRK1A shRNAs and DYRK1A inhibitor harmine, miR-204-5p mimic failed to upregulate the expression of ER stress-related proteins, including Grp78, IRE1α, CHOP, and active caspase-12, in primary cultured dopaminergic neurons. **(C)** The miR-204-5p mimic-induced decrease in protein expression of autophagy markers, including Beclin-1, Atg7, Atg16L1, and LC3-II/I ratio, in primary cultured dopaminergic neurons was prevented by cotransfection of shRNA of DYRK1A or treatment of DYRK1A inhibitor harmine. **(D)** Knockdown of DYRK1A expression or treatment of harmine in primary cultured dopaminergic neurons attenuated miR-204-5p mimic-induced activation of JNK, c-Jun, caspase-9, and caspase-3 in primary cultured SN dopaminergic neurons. **(E)** In the presence of DYRK1A shRNA or DYRK1A inhibitor harmine, the miR-204-5p mimic failed to significantly upregulate the mRNA level of Bim in primary cultured dopaminergic neurons. Each qRT-PCR experiment was performed in triplicate. **(F)** Cotransfection of DYRK1A shRNA or treatment of DYRK1A inhibitor harmine reversed miR-204-5p mimic-induced cell death of primary cultured dopaminergic neurons. For the assays of cell viability, each experiment was performed in triplicate. Each bar indicates the mean ± SEM value of four independent experiments. ^∗^*p* < 0.05, ^∗∗^*p* < 0.01 compared to scramble miR control (SC)-transfected primary cultured dopaminergic neurons.

Transfecting SH-SY5Y cells with shRNAs of DYRK1A significantly decreased the protein level of DYRK1A (Figure 7A). Cotransfection of the shRNA of DYRK1A or treatment of the DYRK1A inhibitor harmine (1μM) reversed miR-204-5p mimic-induced upregulated protein expression of α-Syn, phospho-α-Syn, tau, or phospho-tau in SH-SY5Y dopaminergic cells or primary cultured dopaminergic neurons (Figures 7B, 8A). Knockdown of DYRK1A expression or DYRK1A inhibitor harmine attenuated miR-204-5p mimic-induced upregulation of ER stress markers, including Grp78, PERK, IRE1α, CHOP, and active caspase-12, in SH-SY5Y cells or primary SN dopaminergic neurons (Figures 7C, 8B). In the presence of the shRNAs of DYRK1A or DYRK1A inhibitor harmine, the miR-204-5p mimic failed to decrease expressions of autophagy markers, including Beclin-1, Atg7, Atg16L1, and LC3-II/I ratio, in SH-SY5Y dopaminergic cells or primary cultured dopaminergic neurons (Figures 7D, 8C). Cotransfection of the shRNA of DYRK1A or treatment of harmine reversed miR-204-5p mimic-induced upregulation of JNK, c-Jun, active caspase-9, and active caspase-3 in SH-SY5Y cells or primary SN dopaminergic neurons (Figures 7E, 8D). In the presence of DYRK1A shRNAs or DYRK1A inhibitor harmine, transfection of the miR-204-5p mimic did not significantly upregulate the mRNA expression of proapoptotic Bim in SH-SY5Y dopaminergic cells or primary cultured dopaminergic neurons (Figures 7F, 8E). Knockdown of DYRK1A expression or treatment of harmine also attenuated miR-204-5p mimic-induced cell death of SH-SY5Y cells or primary cultured dopaminergic neurons (Figures 7G, 8F).

## Discussion

MicroRNAs are small non-coding RNAs and downregulate or upregulate the mRNA level by binding to the 3′UTR of the target gene (Bartel, 2004). Dysregulated expression of miRs was identified in body fluids and brain tissues from patients affected with various neurodegenerative disorders, such as AD, PD, and HD (Basak et al., 2016; Leggio et al., 2017; Khodadadian et al., 2018). Therefore, dysregulated levels of miRs can be used as biomarkers of AD, HD, and PD (Basak et al., 2016; Viswambharan et al., 2017). The downregulated or upregulated level of miRs is also believed to participate in the pathogenesis of AD, HD, and PD (Basak et al., 2016; Viswambharan et al., 2017). In this study, the levels of 45 brain-enriched miRs mentioned in a previous study (Kim et al., 2007) were evaluated in serum samples from normal subjects and sporadic PD patients. The results of this study showed that the serum level of only one miR, miR-204-5p, was significantly increased in the PD patients. Consistent with our results, the level of miR-204-5p has been shown to be increased in the postmortem striatum of PD patients (Nair and Ge, 2016). Our finding suggests that the upregulated serum level of miR-204-5p could be used as a biomarker of idiopathic PD.

Previous studies reported that aberrant expression of miRs was observed in serum samples from PD patients. The serum levels of miRs, such as miR-19a, miR-19b, miR-29a, and miR-29c, were decreased in sporadic PD patients or PD patients carrying LRRK2 mutations (Botta-Orfila et al., 2014). The expression of miR-195 was increased and the levels of miR-15b, miR-181a, miR-185, and miR-221 were decreased in serum samples from patients with PD (Ding et al., 2016). In the present study, the levels of three miRs, including miR-26a, miR-138, miR-218, were decreased in serum samples from patients with sporadic PD (Supplementary Table 2).

It is possible that an upregulated level of miR-204-5p is involved in the pathogenesis of PD. Consistent with this hypothesis, an increased level of miR-204-5p was observed in the serum and SN of the MPTP mouse model of PD. In this investigation, we further studied the molecular mechanism of upregulated miR-204-5p in the pathogenesis of PD. The presence of Lewy bodies is the pathological characteristic of PD and causes neurotoxicity (Spires-Jones et al., 2017; Sweeney et al., 2017; Wemheuer et al., 2017). Alpha-Syn, phospho-α-Syn, tau, and phospho-tau are the major components of Lewy bodies (Ishizawa et al., 2003). Interestingly, our results demonstrated that the expression of miR-204-5p significantly increased the protein level of α-Syn, phospho-α-Syn^Ser129^, tau, or phospho-tau^Ser202/Thr205^ in SH-SY5Y dopaminergic cells. Induction of ER stress is believed to participate in the neurodegeneration of SNpc dopaminergic cells and pathogenesis of PD (Xiang et al., 2017). Upregulated expression of α-Syn proteins and phospho-α-Syn proteins caused by miR-204-5p is expected to cause activation of ER stress (Wang and Hay, 2015; Ogen-Shtern et al., 2016; Ganguly et al., 2018). In accordance with this hypothesis, expression of the miR-204-5p mimic increased the expression of ER stress markers, including Grp78, PERK, IRE1α, CHOP, and active caspase-12, in SH-SY5Y dopaminergic cells. Overexpression of α-Syn leads to the decrease in LC3-II and impairs macroautophagy (Winslow et al., 2010). Expression of mutant A30P α-Syn in dopaminergic neurons results in the decrease in LC3-II, indicating an impaired autophagic flux (Lei et al., 2019). Overexpression of WT α-Syn or mutant α-Syn in astrocyte cells decreased the level of LC3-II and caused the inhibition of autophagy (Erustes et al., 2018). Therefore, miR-204-5p-induced increase in protein levels of α-Syn and phospho-α-Syn could also result in the impairment of autophagy, which further facilitates ER stress (Winslow et al., 2010). In accordance with our hypothesis, the protein expression of autophagy markers, including Beclin-1, Atg7, Atg16L1, and LC3-II/I ratio, was downregulated in SH-SY5Y dopaminergic cells expressing miR-204-5p. Interestingly, previous studies indicated that miR-204-5p regulates ER stress in vascular endothelial cells and trabecular meshwork cells (Li et al., 2011; Kassan et al., 2017). MiR-204-5p also regulates in the autophagy of renal clear cell carcinoma (Mikhaylova et al., 2012).

Activation of ER stress caused by miR-204-5p could lead to the induction of active caspase-9 and active caspase-3 by triggering the JNK pathway (Peng and Andersen, 2003; Kuan and Burke, 2005). Following the initiation of ER stress, IRE1 activates downstream targets, JNK and c-Jun. Then, active c-Jun induces the activation of cleaved caspase-9 and caspase-3 by increasing the mRNA level of proapoptotic Bim. Consistent with this hypothesis, the expression of miR-204-5p significantly increased the protein expression of active phospho-JNK^Thr183/Tyr185^, active phospho-c-Jun^Ser63^, cleaved caspase-9, or cleaved caspase-3 in SH-SY5Y cells. The mRNA level of Bim was significantly upregulated in SH-SY5Y dopaminergic cells transfected with the miR-204-5p mimic. Furthermore, upregulated protein expressions of active caspase-12, caspase-9, and caspase-3 caused by miR-204-5p led to the death of SH-SY5Y dopaminergic cells. These findings suggest that the upregulated expression of miR-204-5p may be involved in the etiology of idiopathic PD by causing the activation of ER stress.

To understand the molecular pathogenic mechanism underlying miR-204-5p-induced neurotoxicity in SH-SY5Y dopaminergic cells, it is essential to identify the target gene of miR-204-5p. In the present study, our bioinformatic analysis predicted that DYRK1A is a target gene of miR-204-5p. In accordance with this hypothesis, the 3′UTR of DRYK1A contains three binding sites of miR-204-5p. Subsequent studies using dual-luciferase reporter assays and mutant 3′UTRs of DYRK1A suggest that miR-204-5p positively upregulates the mRNA expression of DYRK1A by directly interacting with the 3′UTR of DYRK1A. Expression of miR-204-5p increased the levels of DYRK1A mRNA and DYRK1A protein in SH-SY5Y dopaminergic cells. The upregulated expression of miR-204-5p in the SN of MPTP mouse model of PD was accompanied by a significant increase in levels of DYRK1A mRNA and DYRK1A protein in the SN of MPTP-treated mice. These results further provide the evidence that miR-204-5p upregulates the mRNA and protein levels of DYRK1A.

Interestingly, previous studies showed that DYRK1A induces the phosphorylation of α-Syn or tau and activation of JNK signaling (Kim et al., 2006; Ryoo et al., 2007; Choi and Chung, 2011). Therefore, it is likely that miR-204-5p causes the upregulated expression of phospho-α-Syn or phospho-tau, ER stress, autophagy impairment, and activation of JNK-mediated apoptotic cascade in SH-SY5Y dopaminergic cells by upregulating the mRNA and protein expression of DYRK1A. Consistent with this hypothesis, knockdown of DYRK1A expression mediated by shRNA of DYRK1A or treatment of DYRK1A inhibitor harmine reversed miR-204-5p-induced increase in protein expression of phospho-α-Syn or phospho-tau, ER stress, autophagy impairment, and activation of JNK-mediated apoptotic pathway in SH-SY5Y dopaminergic cells. Similar to SH-SY5Y dopaminergic cells, downregulation or inhibition of DYRK1A also attenuated miR-204-5p-induced activation of ER stress, autophagy dysfunction, and apoptotic cascade in primary cultured dopaminergic neurons. These finding suggests that miR-204-5p causes neurotoxicity of SH-SY5Y dopaminergic cells or primary cultured dopaminergic neurons by increasing mRNA and protein expression of DYRK1A. Our hypothesis is supported by previous studies reporting that upregulated expression of DYRK1A is likely to be involved in the etiology of neurodegenerative disorders, including AD, HD, and PD (Kang et al., 2005; Abbassi et al., 2015; Kay et al., 2016).

In summary, the present study shows that the serum level of miR-204-5p is increased in sporadic PD patients. The expression of miR-204-5p is also increased in the SN and serum of the MPTP PD mouse model. Our findings suggest that an increased level of miR-204-5p causes ER stress and the death of dopaminergic cells by upregulating the mRNA expression of DYRK1A and by targeting the DYRK1A-mediated apoptotic signaling cascade.

## Data Availability

The raw data supporting the conclusions of this manuscript will be made available by the authors, without undue reservation, to any qualified researcher.

## Ethics Statement

Fifty patients affected with sporadic PD and 50 healthy control subjects were enrolled from the Department of Neurology, Chang Gung Memorial Hospital. Institutional Review Board of Chang Gung Memorial Hospital governed this investigation (IRB no. 201601684B0). All participants submitted informed consent.

## Author Contributions

C-CC and H-LW wrote and edited the manuscript. T-HY, R-SC, H-CC, Y-ZH, Y-HW, Y-CC, Yu-CL, and A-JC designed the study. Ya-CL, Y-JC, Y-WL, C-CH, Y-LC, and C-SL performed the experiments. C-CC, T-HY, Y-LC, Yu-CL, C-CH, and H-LW participated in collecting and analyzing the experimental data.

## Conflict of Interest Statement

The authors declare that the research was conducted in the absence of any commercial or financial relationships that could be construed as a potential conflict of interest.
