# Supplementary material for: Upregulated Expression of MicroRNA-204-5p Leads to the Death of Dopaminergic Cells by Targeting DYRK1A-Mediated Apoptotic Signaling Cascade
Source: Front Cell Neurosci. 2019 Sep 13;13:399. doi: 10.3389/fncel.2019.00399 (PMC6753175; doi:10.3389/fncel.2019.00399)
Supplement: Supplementary file 1 [file Table_1.DOCX]

Supplemental Table 1. Demographic data for studying subjects.

|  | Normal subjects | PD |
| --- | --- | --- |
| Sex (Male/Female) | 23/27 | 24/26 |
| Age* | 63.76 ± 1.10 | 66.38 ± 0.95 |
| Age at onset | - | 62.18 ± 0.93 |

Data represent the mean ± SEM

*There is no significant difference between the mean ages of normal subjects and PD patients.
