# Supplementary material for: Upregulated Expression of MicroRNA-204-5p Leads to the Death of Dopaminergic Cells by Targeting DYRK1A-Mediated Apoptotic Signaling Cascade
Source: Front Cell Neurosci. 2019 Sep 13;13:399. doi: 10.3389/fncel.2019.00399 (PMC6753175; doi:10.3389/fncel.2019.00399)
Supplement: Supplementary file 2 [file Table_2.DOCX]

Supplemental Table 2. The levels of miRs in PD serum samples

| MicroRNAs selected for target analysis | Fold change | P value | Forward primer (5'-3') | Universal reverse primer |
| --- | --- | --- | --- | --- |
| hsa-miR-26a-5p | -1.42 | 0.017 | CGGCGGTTCAAGTAATCCAGGA | CAACTGGTGTCGTGGAGTCGG |
| hsa-miR-138-5p | -1.30 | 0.019 | CGGCGGAGCTGGTGTTGTGAAT | CAACTGGTGTCGTGGAGTCGG |
| hsa-miR-204-5p | 2.24 | 0.009 | CGGCGGTTCCCTTTGTCATCCT | CAACTGGTGTCGTGGAGTCGG |
| hsa-miR-218-5p | -1.25 | 0.029 | CGGCGGTTGTGCTTGATCTAAC | CAACTGGTGTCGTGGAGTCGG |
| hsa-miR-224-5p | 2.35 | 0.096 | CGGCGGTCAAGTCACTAGTGGT | CAACTGGTGTCGTGGAGTCGG |
| hsa-miR-101-5p | 1.78 | 0.157 | CGGCGGCAGTTATCACAGTGCT | CAACTGGTGTCGTGGAGTCGG |
| hsa-miR-107 | 1.54 | 0.242 | CGGCGGAGCTTCTTTACAGTGT | CAACTGGTGTCGTGGAGTCGG |
| hsa-miR-124-3p | 0.54 | 0.544 | CGGCGGCGTGTTCACAGCGGAC | CAACTGGTGTCGTGGAGTCGG |
| hsa-miR-125a-5p | 0.53 | 0.556 | CGGCGGTCCCTGAGACCCTTTA | CAACTGGTGTCGTGGAGTCGG |
| hsa-miR-127-5p | -0.74 | 0.174 | CGGCGGCTGAAGCTCAGAGGGC | CAACTGGTGTCGTGGAGTCGG |
| hsa-miR-128-2-5p | 1.23 | 0.331 | CGGCGGGGGGGCCGATACACTG | CAACTGGTGTCGTGGAGTCGG |
| hsa-miR-130a-5p | 0.93 | 0.332 | CGGCGGGCTCTTTTCACATTGT | CAACTGGTGTCGTGGAGTCGG |
| hsa-miR-133b | 0.74 | 0.449 | CGGCGGTGGCTGGTCAAACGGA | CAACTGGTGTCGTGGAGTCGG |
| hsa-miR-137-5p | -0.35 | 0.668 | CGGCGGACGGGTATTCTTGGGT | CAACTGGTGTCGTGGAGTCGG |
| hsa-miR-142-3p | -0.62 | 0.385 | CGGCGGTGTAGTGTTTCCTACT | CAACTGGTGTCGTGGAGTCGG |
| hsa-miR-142-5p | -0.61 | 0.284 | CGGCGGCATAAAGTAGAAAGCA | CAACTGGTGTCGTGGAGTCGG |
| hsa-miR-143-5p | -0.13 | 0.784 | CGGCGGGGTGCAGTGCTGCATC | CAACTGGTGTCGTGGAGTCGG |
| hsa-miR-152-5p | -0.25 | 0.556 | CGGCGGAGGTTCTGTGATACAC | CAACTGGTGTCGTGGAGTCGG |
| hsa-miR-153-5p | -0.34 | 0.357 | CGGCGGTCATTTTTGTGATCTG | CAACTGGTGTCGTGGAGTCGG |
| hsa-miR-15a-5p | 0.52 | 0.656 | CGGCGGTAGCAGCACATAATGG | CAACTGGTGTCGTGGAGTCGG |
| hsa-miR-15b-5p | 0.46 | 0.679 | CGGCGGTAGCAGCACATCATGG | CAACTGGTGTCGTGGAGTCGG |
| hsa-miR-16-5p | 0.45 | 0.720 | CGGCGGTAGCAGCACGTAAATA | CAACTGGTGTCGTGGAGTCGG |
| hsa-miR-17-3p | -0.77 | 0.129 | CGGCGGACTGCAGTGAAGGCAC | CAACTGGTGTCGTGGAGTCGG |
| hsa-miR-17-5p | -0.25 | 0.938 | CGGCGGCAAAGTGCTTACAGTG | CAACTGGTGTCGTGGAGTCGG |
| hsa-miR-184 | -0.20 | 0.888 | CGGCGGCCCTTATCACTTTTCC | CAACTGGTGTCGTGGAGTCGG |
| hsa-miR-191-5p | -0.40 | 0.355 | CGGCGGCAACGGAATCCCAAAA | CAACTGGTGTCGTGGAGTCGG |
| hsa-miR-19a-5p | -0.99 | 0.353 | CGGCGGAGTTTTGCATAGTTGC | CAACTGGTGTCGTGGAGTCGG |
| hsa-miR-210-5p | 0.43 | 0.767 | CGGCGGAGGGCAGCCCCTGCCC | CAACTGGTGTCGTGGAGTCGG |
| hsa-miR-212-5p | 0.43 | 0.845 | CGGCGGACCTTGGCTCTAGACT | CAACTGGTGTCGTGGAGTCGG |
| hsa-miR-23a-5p | 0.33 | 0.880 | CGGCGGGGGGTTCCTGGGGATG | CAACTGGTGTCGTGGAGTCGG |
| hsa-miR-24-3p | 0.21 | 0.987 | CGGCGGTGCCTACTGAGCTGAT | CAACTGGTGTCGTGGAGTCGG |
| hsa-miR-25-5p | 0.00 | 0.999 | CGGCGGAGGCGGAGACTTGGGC | CAACTGGTGTCGTGGAGTCGG |
| hsa-miR-26b-5p | 0.00 | 0.998 | CGGCGGTTCAAGTAATTCAGGA | CAACTGGTGTCGTGGAGTCGG |
| hsa-miR-27a-5p | 0.00 | 0.995 | CGGCGGAGGGCTTAGCTGCTTG | CAACTGGTGTCGTGGAGTCGG |
| hsa-miR-29c-5p | -0.96 | 0.202 | CGGCGGTGACCGATTTCTCCTG | CAACTGGTGTCGTGGAGTCGG |
| hsa-miR-30a-3p | -0.98 | 0.211 | CGGCGGCTTTCAGTCGGATGTT | CAACTGGTGTCGTGGAGTCGG |
| hsa-miR-30a-5p | -0.44 | 0.778 | CGGCGGTGTAAACATCCTCGAC | CAACTGGTGTCGTGGAGTCGG |
| hsa-miR-320a-5p | -0.43 | 0.847 | CGGCGGGCCTTCTCTTCCCGGT | CAACTGGTGTCGTGGAGTCGG |
| hsa-miR-330-5p | -0.57 | 0.570 | CGGCGGTCTCTGGGCCTGTGTC | CAACTGGTGTCGTGGAGTCGG |
| hsa-miR-331-5p | -0.56 | 0.568 | CGGCGGCTAGGTATGGTCCCAG | CAACTGGTGTCGTGGAGTCGG |
| hsa-miR-34a-5p | -0.53 | 0.642 | CGGCGGTTGGCAGTGTCTTAGC | CAACTGGTGTCGTGGAGTCGG |
| hsa-miR-7-5p | -0.51 | 0.640 | CGGCGGTGGAAGACTAGTGATT | CAACTGGTGTCGTGGAGTCGG |
| hsa-miR-93-5p | -0.48 | 0.984 | CGGCGGCAAAGTGCTGTTCGTG | CAACTGGTGTCGTGGAGTCGG |
| hsa-miR-99a-5p | -0.53 | 0.294 | CGGCGGAACCCGTAGATCCGAT | CAACTGGTGTCGTGGAGTCGG |
